# Endoscopic reduction of a volvulus of the sigmoid colon in pregnancy: case report and a comprehensive review of the literature

**DOI:** 10.1186/1749-7922-9-41

**Published:** 2014-06-30

**Authors:** Zia Aftab, Adriana Toro, Abdelrahmam Abdelaal, Mohammed Dasovky, Salahdin Gehani, Abdelatif Abdel Mola, Isidoro Di Carlo

**Affiliations:** 1 Department of Surgery, Hamad Medical Corporation, Doha, Qatar; 2 Department of Surgery, Taormina Hospital, Taormina, Italy; 3 Department of Surgical Sciences, Organ Transplantation, and Advanced Technologies, University of Catania, Cannizzaro Hospital, Via Messina 829, Catania 95126, Italy

**Keywords:** Sigmoid volvulus, Pregnancy, Endoscopic reduction

## Abstract

Sigmoid volvulus is a rare, but serious, complication that can occur during pregnancy. We present a case of a 33-year-old pregnant female in the third trimester with a sigmoid volvulus. Detorsion of the volvulus was performed during colonoscopy. The patient underwent an elective sigmoidectomy at a later date. Prompt diagnosis of the volvulus sigmoid is critical to minimize fetal and maternal morbidity and mortality. Sigmoidoscopic detorsion or surgical resection are the treatment options, depending on bowel viability. A review of the literature was done.

## Introduction

Intestinal obstruction in pregnancy is uncommon with a reported incidence ranging from one in 1500 to one in 66,431 [1]. The most common causes of intestinal obstruction in pregnancy are adhesion, intestinal volvulus, intussusception, carcinoma, hernia and appendicitis [2]. In 1885, Braun was the first surgeon to describe a case of sigmoid volvulus during pregnancy [3]. Intestinal obstruction due to sigmoid volvulus during pregnancy remains extremely rare and is of extreme gravity especially if not recognized and treated precociously [4].

The clinical presentation is similar to that in non-pregnant females, but is masked by the enlarged uterus and the physiological changes of pregnancy. The sigmoid volvulus occurs when the sigmoid colon wraps around itself and its mesentery. The increasing size of the uterus may elevate a mobile sigmoid colon from the pelvis and produce a partial obstruction either due to pressure or kinking of this portion of the bowel [2].

This difficult presentation, along with a delay in diagnosis, is the main reason behind the high morbidity and mortality of this condition. Outcomes may include bowel ischemia, necrosis, gangrene, perforation, peritonitis, preterm delivery and both fetal and maternal death [5].

In this report, we present a patient diagnosed with sigmoid volvulus during pregnancy who was initially treated non-operatively by detorsion with flexible endoscopy and underwent elective resection of the sigmoid colon after delivery. We also undertook a comprehensive review of the literature.

## Case presentation

A 33-year-old female of 32 weeks’ gestation, para 2 gravida 3, presented with generalized abdominal pain of 2 days’ duration. The pain was gradually increasing in intensity, colicky in nature and not associated with vomiting, fever or anal bleeding. On the second day, it was mainly felt in the right and left lower quadrants with abdominal distension. She passed flatus until 8 h prior to presentation, after which she was completely constipated. The patient related this symptom to her pregnancy, but as her symptoms did not improve she presented to Gynecological and Obstetric emergency department.

The patient had no significant medical history, except two previous cesarean sections (the last one 5 years ago).

On clinical examination she was afebrile, her pulse rate was 100, blood pressure 120/80 mmHg and oxygen saturation 99%. Her abdomen was distended and soft with mild tenderness mainly over the left iliac fossa, and palpable bowel loop in the upper abdomen. Bowel sounds were audible but sluggish. Her gravid uterus corresponded to 32 weeks’ gestation. Anal examination showed no fissure or prolapsed piles. Stools with no blood were found in the rectum.

Fetus viability was assessed by the gynecologist, and was normal and alive.

Routine laboratory studies were significant only for an elevated white blood cell count of 12.4 K/æL, which could have been due to normal physiological response in pregnancy.

**Figure 1:**
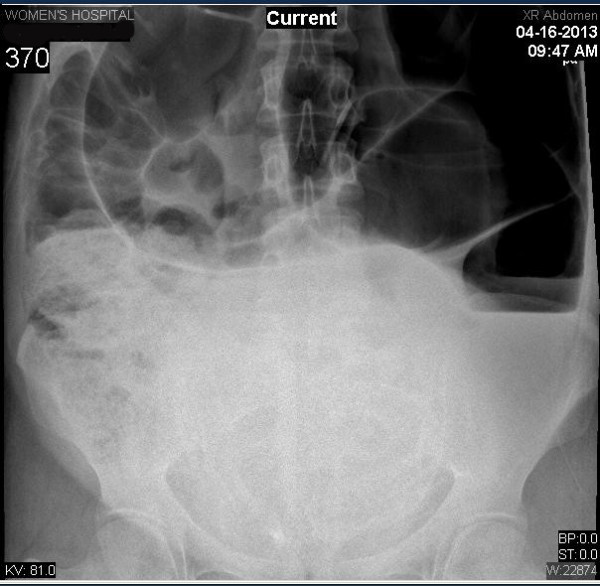
X-ray examination showing the enormous dilatation and complete occlusion of the sigmoid.

The hemoglobin was 11.6 g/dL and platelets were 183 K/æL. The electrolytes and liver function test were normal.Thorax and cardio examinations were within normal. Abdominal x-ray showed severely distended bowel loops with multiple air fluid levels with no air seen in the rectum (Figure 1).

Based on the patient’s presentation and examination, a diagnosis of intestinal obstruction was reached (sigmoid volvulus was highly suspected). A nasogastric tube (NGT) and Foley catheter were inserted. The gastroenterology team was consulted and it was decided to take the patient for emergency sigmoidoscopy.

This revealed a sigmoid volvulus that was derotated and deflated. The scope was passed until the splenic flexure. A 20 French rectal tube was inserted with no immediate complications.

The patient was transferred to the high-dependency unit for close monitoring. On subsequent assessment, she was not in pain, with resolution of the abdominal distension and passage of flatus. She was afebrile, with a pulse rate of 90 and blood pressure of 120/70 mmHg. An abdominal x-ray the day after showed no distended bowel loops or fluid levels. The NGT was removed.

**Figure 2:**
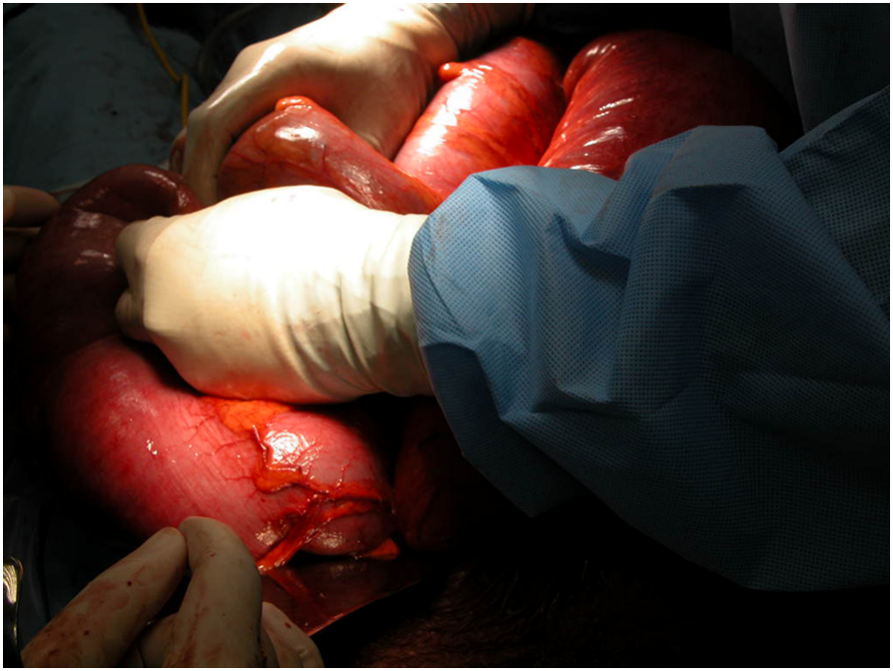
Dilatation of the sigmoid during the cesarean section delivery.

She was started on fluids until she could tolerate a full diet. The rectal tube was removed 2 days after the procedure and the patient was passing normal peristalsis. She was shifted back to the ward and kept for 2 more days for observation of fetal well-being, after which she was discharged for follow-up in the surgical and gynecology outpatients’ departments.Later she presented to gynecology for an elective cesarean section. During surgery a hugely distended sigmoid colon was found. Preoperative colonic detorsion was done and elective sigmoidectomy planned (Figure 2).

### Literature review methodology

A literature search was performed using MEDLINE (PubMed), Google Scholar and The Cochrane Library, and articles from January 1900 until June 2013, edited in Italian, English and French, were analyzed. The keywords used were: “sigmoid volvulus,” “pregnancy”. These keywords were added alone or in combination using the Boolean operator “AND”. Only patients with sigmoid volvulus in pregnancy were considered for the review. Irrelevant articles evident from the title and abstract were excluded. Relevant articles referenced in these publications were obtained and the “related article” function was used to widen the results.

**Figure 3:**
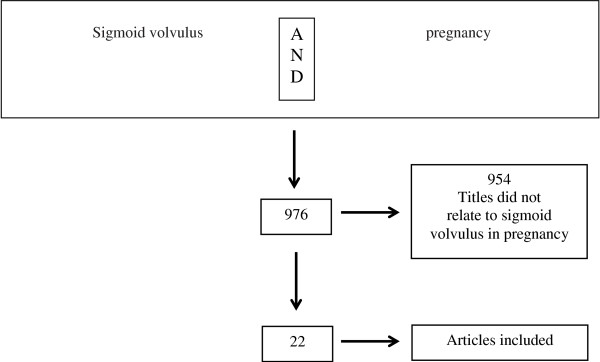
Flowchart describing the selection of studies included in this article.

The search initially yielded 976 articles (Figure 3). After screening titles, 954 articles were excluded because they were not related to sigmoid volvulus in pregnancy. A total of 22 articles were found for the present study [1-4,6-23] and a total of 95 patients were analyzed.

## Discussion

Intestinal obstruction during pregnancy, which was first reported by Houston in 1830, is a rare complication with an incidence ranging from one in 1500 to one in 66,431 deliveries [5]. It can be caused by many factors including congenital or postoperative adhesions, volvulus, intussusceptions, colonic masses, hernia and appendicitis [5]. In relation to the literature, the sigmoid volvulus represents the commonest cause of intestinal obstruction during pregnancy, occurring at rates between 3.1% and 12.5% depending on the series [24,25]. Table 1 shows all 95 cases of sigmoid volvulus reported in the literature worldwide [1-4,6-23].

**Table 1:** Reported cases of sigmoid volvulus in pregnancy until 2013

**Authors**	**Year**	**Cases**	**Gestational age (weeks)**	**Duration of symptoms (hours)**	**Outcome**	
					**Mother**	**Fetus**
Lambert AC [6]	Before 1931	29	--	--	--	--
Kohn SG [7]	1931-1944	12	--	--	--	--
Harer WB Jr [2]	1944-1958	11	--	--	--	--
Lazaro EJ [8]	1958-1969	13	--	--	--	--
Fraser JL [9]	1983	1	32	24	Healthy	Alive
Hofmeyr GJ [10]	1985	2	33	72	Healthy	IUD
			26	72	Expired	IUD
Keating JP [11]	1985	1	34	24	Healthy	Alive
Allen JC [12]	1990	1	28	24	Healthy	Alive
Lord SA [1]	1996	1	36	24	Healthy	Alive
Joshi MA [13]	1999	1	28	24	Healthy	IUD
De U [14]	2005	1	24	72	Healthy	IUD
Alshawi JS [15]	2005	1	28 and 35	24	Healthy	Alive
Iwamoto I [4]	2007	1	35	72	Expired	IUD
Vo TM [16]	2008	1	28	24	Healthy	Alive
Narjis Y [17]	2008	1	24	--	Healthy	Alive
Kolusari A [18]	2009	3	7	24	Healthy	Alive
			31	48	Healthy	IUD
			32	48	Healthy	Alive
Machado NO [19]	2009	1	18	18	Expired	Alive
Togo A [20]	2011	1	25	48	Expired	Alive
Khan MR [21]	2012	1	30	144	Expired	IUD
Atamanalp SS [22]	2008	9	3rd trimester	24	Healthy	--
			2nd trimester	36	Healthy	--
			3rd trimester	72	Expired	--
			3rd trimester	20	Healthy	--
			3rd trimester	24	Healthy	--
			2nd trimester	36	Healthy	--
			3rd trimester	12	Healthy	--
			1st trimester	22	Healthy	--
			3rd trimester	18	Healthy	--
Dray X [23]	2012	1	37	12	Expired	Alive
Nascimento EFR [3]	2012	1	33	72	Expired	IUD
This article	2013	1	32	48	Healthy	Alive

Sigmoid volvulus occurs more commonly in pregnant than in non-pregnant women and affects mainly chronically constipated patients with a long redundant sigmoid colon [24]. High-fiber diets are also a predisposing factor [25].

The mechanism of sigmoid volvulus in pregnancy has been explained as being caused by displacement of an abnormally mobile sigmoid colon by the enlarging uterus. This causes the colon to rise out of the pelvis and twist around the fixation point on the sigmoid colon and its mesocolon. This mechanism may lead to mechanical obstruction and vascular compromise of the bowel [24], and explains the increased incidence of sigmoid volvulus in the third trimester.

The mean duration of symptoms for pregnancy patients in the literature is 40.6 h [1-4,6-23] with a range from 1 h to 6 days [1-4,6-23]. Our patient presented at our hospital approximately 48 h from the onset of intestinal obstructive symptoms [5].

A typical symptom of intestinal obstruction is intermittent colicky abdominal pain, which gradually progresses with nausea and clear initial vomiting progressing to fecal vomiting.

Furthermore, the patient may present with fever, dehydration, absence of bowel sound and leukocytosis. These clinical signs might easily be detected in a non-pregnant woman, but are common in pregnancy [16].

The delay in diagnosis of sigmoid volvulus may lead to bowel infarction and necrosis with hypovolemia, electrolyte disturbances, renal failure, metabolic acidosis, septic shock and multiple organ failure with a significant devastating outcome for the mother and the fetus.

Maternal mortality for sigmoid volvulus has been reported to be 5% if the bowel is viable, but rises to over 50% if perforation has occurred [13]. Fetal mortality in sigmoid volvulus is approximately 30%. The fetal death could be caused by reduction in placental blood flow in hypovolemia, or by reduction of the abdominal and pelvic blood flow due to increased intraabdominal pressure as a result of massive sigmoid dilatation [10].

Diagnosis of intestinal obstruction in pregnancy is difficult, as the classical symptoms of abdominal distension, nausea and vomiting are common in uncomplicated pregnancies [13]. The diagnosis should be suspected when a pregnant woman presents with a clinical symptom of abdominal pain, distention and absolute constipation [5]. The leukocytosis can be a consistent sign but in the first phase of the disease can be normal or slightly elevated [15]. Furthermore, the white cell count is normally elevated in pregnancy [22].

The use of radiological tools can be useful to establish the diagnosis, but many clinicians are reluctant to use them for fear of fetal complications. Radiation exposure may lead to chromosomal abnormalities, neurologic mutations and increased risk of hematologic malignancies [26]. However, even with plain computed tomography (CT) scans of the abdomen, the radiation dose is still thought to be within the safe exposure limit (5–10 rads) [27]. Still, many authors believe it is best avoided because of the radiation risks to the fetus. In contrast, abdominal and obstetric ultrasonography may eliminate the radiologic risk and provide information about the fetus [22].

The management of sigmoid volvulus in pregnancy requires a multidisciplinary approach with general surgeons, obstetricians, and neonatologists [16].

The patient should be treated with fluids, electrolyte balance correction, prophylactic antibiotics, and nasogastric decompression. Tocolytics should be administered if uterine irritability is observed, and steroids initiated to promote fetal lung maturity [22]. Obstetric intervention should strictly depend on the condition of the fetus.

The integrity of the uterus has to be preserved in the case of a vital fetus [19]. In cases of fetal maturity, a vaginal labor can be induced if the condition of the mother and fetus is stable [19]. If cesarean section is needed, the sigmoid resection can follow with particular attention to avoid disruption of the sigmoid colon and relative contamination of the uterus.

Cesarean section is suggested by some authors also in case of fetal death. In such cases, this procedure must be done first and special care taken to avoid contamination of the peritoneum. Indeed, this can itself be a cause of mortality due to a consequent severe puerperal infection [13].

A delay in diagnosis and surgical intervention over 48 h can have a significant impact on the ultimate outcome of the mother and fetus [2]. The management of sigmoid volvulus in pregnancy begins with aggressive hydration and proximal bowel decompression [13]. In the absence of mucosal ischemia, sigmoidoscopic detorsion and rectal tube insertion is possible. In recurrent cases, elective sigmoidectomy can be safely performed in the second trimester [20]. Otherwise, surgery can be postponed until after delivery.

In cases of bowel gangrene or perforation, prompt surgical intervention through a midline laparotomy is essential. Thorough peritoneal lavage of the resection of the necrotic bowel segments is mandatory. This is followed by either primary anastomosis or stoma formation (Hartman’s procedure) [28].

The prognosis of sigmoid volvulus in pregnancy is poor. In the last century, the maternal mortality rate was 21–60% and fetal mortality rate was 50% [5]. In recent decades, the maternal mortality has decreased to 6–12% and fetal mortality to 20–26% [29]. The major causes of maternal mortality are toxic and/or hypovolemic shock, whereas impairment of placental blood flow due to increased intraabdominal pressure affects fetal mortality [30].

## Conclusion

Sigmoid volvulus is a rare and potentially fatal condition in pregnancy that requires a multidisciplinary approach with general surgeons, obstetricians, and neonatologists. Prompt diagnosis is critical for early management, to minimize fetal and maternal morbidity and mortality. Abdominal pain may be the only findings, and sigmoidoscopic detorsion or surgical resection are the treatment options, depending on bowel viability.

## Consent

Written informed consent was obtained from the patient for publication of this Case report and any accompanying images.

## Competing interests

The authors declare that they have no competing interests.

## Authors’ contributions

ZA, IDC: Have made substantial contributions to conception and design. SG, AAM: acquisition of data. AA, MD: analysis and interpretation of data. AT, ZA: have been involved in drafting the manuscript. IDC: revising it critically for important intellectual content. AA, MD, SG, AAM: have given final approval of the version to be published. ZA: agree to be accountable for all aspects of the work in ensuring that questions related to the accuracy or integrity of any part of the work are appropriately investigated and resolved. All authors read and approved the final manuscript.
